# PdGa nanoalloys loaded on single atom Co dispersed nitrogen doped carbon for ethanol electrooxidation: improved C1 pathway selectivity and durability

**DOI:** 10.1039/d5sc05140a

**Published:** 2025-09-23

**Authors:** Chengming Huang, Xia Chen, Wenjing Zhang, Fangzheng Wang, Yunchuan Tu, Jing Li, Zidong Wei

**Affiliations:** a State Key Laboratory of Advanced Chemical Power Sources, School of Chemistry and Chemical Engineering, Chongqing University 401331 Chongqing China yctu@cqu.edu.cn lijing@cqu.edu.cn zdwei@cqu.edu.cn

## Abstract

Direct ethanol fuel cells (DEFCs) are among the most efficient and environmentally friendly energy conversion devices, and the development of ethanol oxidation reaction (EOR) catalysts with high C1 pathway selectivity and electrochemical durability is essential for the commercialization of DEFCs. In this study, we demonstrate that engineering a single-atom Co dispersed support is an effective strategy for modulating the electronic structure of supported PdGa alloy nanoparticle catalysts, thereby significantly enhancing the EOR performance. In an alkaline system, PdGa/Co_SA_-NC exhibits a C1 path selectivity of 34.3% at 0.8 *V*_RHE_, being 2.3 and 7.0 times those of PdGa/NC and commercial Pd/C, respectively. Also, in the accelerated durability testing PdGa/Co_SA_-NC well retains 74.2% and 35.0% of the initial activity after 1000 and 2500 cycles, respectively. Our theoretical calculations show that PdGa alloy nanoparticles exhibit significantly enhanced electron transfer to the Co_SA_-NC support compared to their PdGa/NC counterpart, leading to a substantial increase in charge density at the interfacial region of PdGa/Co_SA_-NC. The well modified electron structures optimize the adsorption energies of key intermediate species on Pd sites, simultaneously promoting both C–C bond cleavage and *CO oxidation, thus enhancing the C1 pathway selectivity and EOR durability on PdGa/Co_SA_-NC.

## Abstract 2

Single-atom Co dispersion support engineering effectively tunes the electronic structure of PdGa alloy nanoparticle catalysts, delivering excellent EOR performance with high activity and selectivity toward the C1 pathway.

## Graphical abstract

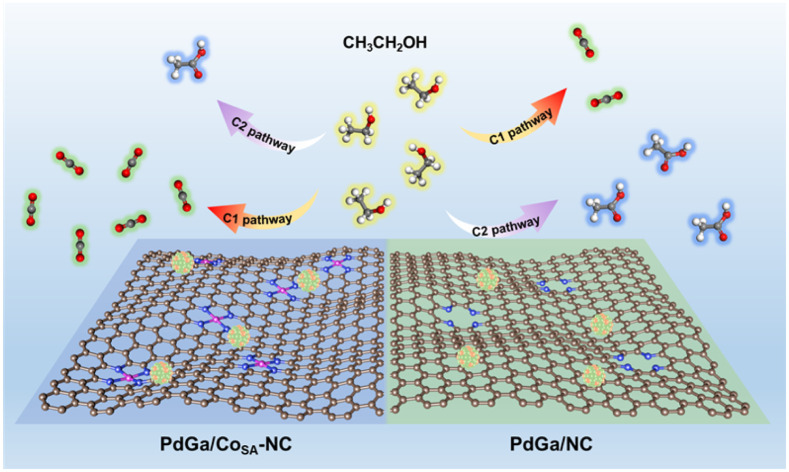

## 1 Introduction

Direct ethanol fuel cells (DEFCs) have been recognized as one of the most promising energy power units for portable and mobile applications due to their high energy density, convenient storage/transportation and environmental friendliness.^1–4^ It is generally accepted that the ethanol oxidation reaction (EOR) follows the C1/C2 dual pathway reaction mechanism.^5–8^ And the incomplete oxidation of ethanol to acetic acid or acetate products (C2 pathway), with only 4 e^−^ transfer, leads to the incomplete utilization of ethanol fuel and greatly reduces the energy conversion efficiency. In contrast, the complete oxidation of ethanol to CO_2_ (C1 pathway) can maximize the energy release due to the transfer of 12 e^−^. However, the high energy barrier of C–C bond cleavage and the slow oxidation of various carbonaceous intermediates such as *CH_*x*_ and *CO, severely hinder the kinetics of the C1 pathway.^9–12^ Therefore, the synthesis of EOR catalysts that is able to accelerate the C1 pathway process, inhibit the C2 process, and thus enhance the C1 selectivity is crucial for practical DEFCs.

Presently, noble metal Pd has been widely recognized as the most active catalyst for the EOR in alkaline electrolytes,^13–17^ and to enhance its catalytic performance, the approach of alloying Pd with a secondary metal has proven to be an effective route.^18–20^ In general, the enhanced catalytic properties of Pd alloys can be attributed to two main aspects: the second metal can change the electronic structure of Pd, thus promoting the C–C bond breaking for higher C1 pathway selectivity; on the other hand, the second component in the alloys can accelerate *OH adsorption, which facilitates the removal of *CH_*x*_ and *CO species and then improves reaction kinetics and the anti-poisoning capability of the catalyst.^21–28^ However, due to the complexity of the EOR process, the acquired C1 pathway selectivity and the overall catalytic performance remain significantly below the anticipated levels. Consequently, there is an urgent need to develop innovative catalyst design strategies to substantially enhance the various metrics of EOR performance. A few recent reports have indicated that the construction of specific external sites can well modify the electronic structure of nanoalloys, and one of the practically effective ways is the adoption of a support material containing singly-dispersed atoms.^9,29–33^ However, the research in this specific field is still limited, and the efficacy of such regulation in the electrocatalytic EOR has yet to be substantiated.

Herein, we choose metallic PdGa alloy nanoparticles as the major catalytic sites as they well facilitate the complete electrooxidation of ethanol due to the p–d orbital hybridization effect.^34^ Then by loading PdGa alloy nanoparticles on single Co atom dispersed N-doped carbon (Co_SA_-NC), we can investigate the enhancing effect of the atomic level support on the catalytic PdGa alloy centers. Interestingly the prepared PdGa/Co_SA_-NC catalyst achieves highly efficient EOR catalysis in an alkaline electrolyte as the mass activity reaches 6.93 A mg_Pd_^−1^, being 2.1 and 5.6 times those of PdGa/NC (3.28 A mg_Pd_^−1^) and Pd/C (1.24 A mg_Pd_^−1^), respectively. Also, it exhibits a C1 path selectivity of 34.3% at 0.8 *V*_RHE_, being 2.3 and 7.0 times those of PdGa/NC and commercial Pd/C, respectively. And in the accelerated durability testing, PdGa/Co_SA_-NC well retains 74.2% and 35.0% of the initial activity after 1000 and 2500 cycles, respectively, exceeding the performances of state-of-the-art materials. Density Functional Theory (DFT) calculations show that PdGa alloy nanoparticles exhibit significantly enhanced electron transfer to the Co_SA_-NC support compared to their PdGa/NC counterpart, leading to a substantial increase in charge density at the interfacial region of PdGa/Co_SA_-NC. The well modified electron structures optimize the adsorption energies of key intermediate species on Pd sites, simultaneously promoting both C–C bond cleavage and *CO oxidation on PdGa/Co_SA_-NC, which not only enhances the C1 pathway selectivity but also confers superior EOR durability through significantly improved CO tolerance. This study demonstrates that engineering single-atom dispersed supports represents an effective strategy for modulating the electronic structure of supported alloy catalysts, thereby significantly enhancing their electrocatalytic performance. The fundamental understanding provides a valuable framework for the rational design of highly efficient catalysts for various crucial electrocatalytic processes.

## 2 Results and discussion

### 2.1 Characterization of catalysts

**Fig. 1:**
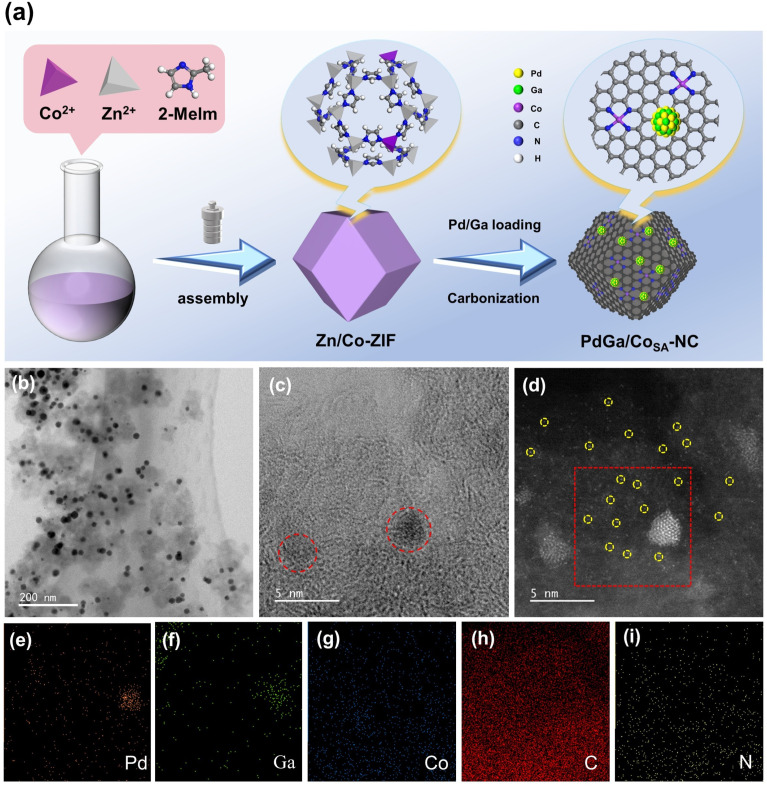
Synthesis and characterization of PdGa/Co_SA_-NC. (a) Schematic illustration of the synthetic process for PdGa/Co_SA_-NC; (b and c) TEM images of PdGa/Co_SA_-NC; (d–i) HAADF-TEM image and corresponding elemental maps of PdGa/Co_SA_-NC.

The synthesis process of PdGa/Co_SA_-NC is schematically illustrated in Fig. 1a and the experimental details are given in the SI. Briefly, the metal–organic framework material Zn/Co-ZIF was first prepared by introducing a small amount of metallic Co into ZIF-8 during the assembly of the latter.^35,36^ After Pd^2+^ and Ga^3+^ were adsorbed on the surface of Zn/Co-ZIF, the obtained composite was calcined at 900 °C under H_2_/Ar flow to form the final product PdGa/Co_SA_-NC. During this annealing process, Zn species is evaporated at ∼700 °C, which promotes Co–N bonding, and formation of the single atom Co dispersed nitrogen-doped carbon Co_SA_-NC, as displayed in Fig. S1. The transmission electron microscopy (TEM), HAADF-STEM and EDX mapping images of PdGa/Co_SA_-NC (Fig. 1b–i and S2) show that PdGa alloy particles possess a quite narrow size distribution of 14.0 nm, which together with singly dispersed Co atoms are evenly anchored on the NC support. To illustrate the role of atomic Co, we also prepared a Co-free control sample PdGa/NC by a similar method, except that no Co precursor was used (Fig. S3). Table S1 gives the composition of the two materials measured by using an Inductively Coupled Plasma Optical Emission Spectrometer (ICP-OES) which shows that PdGa/Co_SA_-NC and PdGa/NC possess quite similar Pd content of 3.9 wt% and Ga content of 3.4 wt%, and Zn species is almost non-existent in both.

**Fig. 2:**
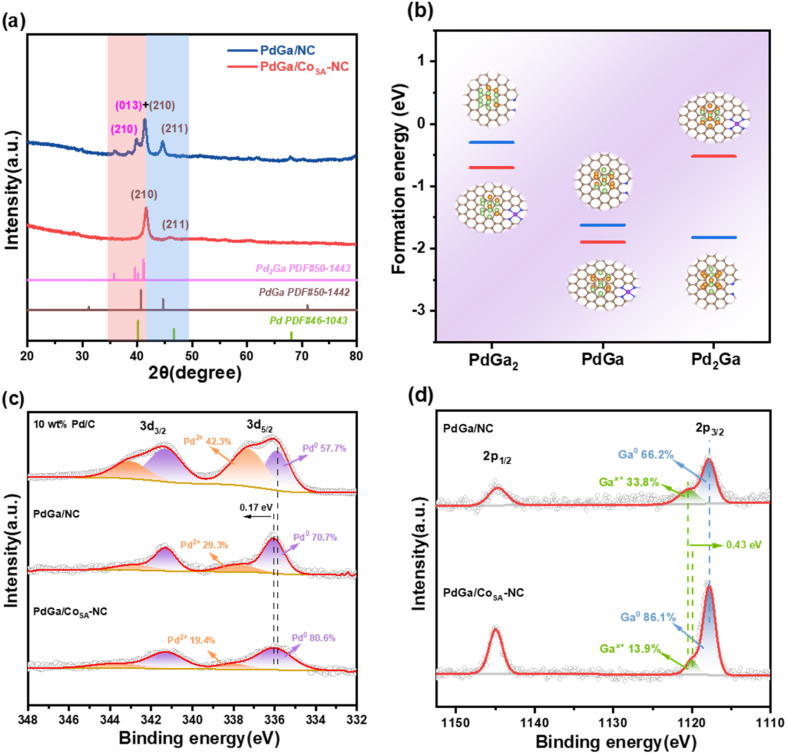
Structural characterization of the as-prepared catalysts. (a) XRD patterns; (b) formation energies of PdGa_2_, PdGa and Pd_2_Ga on Co_SA_-NC or N–C; (c) Pd 3d XPS spectra; (d) Ga 2p XPS spectra.

Fig. 2a shows the X-ray diffraction (XRD) patterns. PdGa/Co_SA_-NC displays two distinct diffraction peaks at 2*θ* of 41.6° and 46.0°, which correspond to the (210) and (211) crystal planes of the PdGa alloy (no. 50-1442); both shift 1.2° to the higher 2*θ* direction. Consideration of the mapping results in Fig. 1g shows that a Co signal does not arise in the PdGa alloy position; the alloy composition is determined to be PdGa instead of PdGaCo, and the peak shift might be due to the lattice shrinkage induced by the strong interaction between alloy particles and the Co_SA_-NC support.^37,38^ As listed in Table S2, the lattice parameters of PdGa/Co_SA_-NC and PdGa/NC catalysts are compared.^39,40^ The results indicate that, relative to the NC support, the formation of the Co_SA_-NC support induces a compressive lattice strain of 0.69% in the PdGa alloy. Besides, no metallic cobalt peaks were detected, confirming the absence of cobalt nanoparticle formation, which is primarily attributed to the low Co : Zn atomic ratio (1 : 99) in the precursors. In comparison, PdGa/NC gives multiple diffraction peaks, and the two located at 41.4° and 44.7° match well with those of PdGa. Besides, the peak located at 41.4°, as well as the two located at 39.7° and 35.8°, can be well attributed to the (013), (210), and (112) crystal planes of Pd_2_Ga alloy (no. 50-1443), respectively. This observation shows that there exists a mixed phase of PdGa and Pd_2_Ga in PdGa/NC. This conclusion is further validated by Selected Area Electron Diffraction (SAED) analysis, as shown in Fig. S4. For PdGa/Co_SA_-NC, diffraction patterns corresponding to the (210) and (211) planes of the PdGa alloy were observed. In contrast, PdGa/NC exhibited diffraction patterns from (210) and (211) planes of the PdGa alloy, as well as (112) and (211) planes of the Pd_2_Ga alloy. In particular, the single phase PdGa formed on Co_SA_-NC and the mixed PdGa/Pd_2_Ga phase obtained on NC inspire us to investigate if the single atom Co in the NC support could affect the alloy composition. Using the DFT method, we calculated the formation energies of PdGa_2_, PdGa, and Pd_2_Ga alloy particles on Co_SA_-NC and NC supports (Fig. S5 and S6, SI). As displayed in Fig. 2b, on Co_SA_-NC, the formation energies of PdGa_2_ (−0.70 eV) and Pd_2_Ga (−0.52 eV) are greatly higher than that of PdGa (−1.90 eV), and on NC, the formation energies of PdGa (−1.62 eV) and Pd_2_Ga (−1.82 eV) are significantly lower than that of PdGa_2_ (−0.30 eV), which well indicates that the Co_SA_-NC surface favors the formation of single phase PdGa particles while NC is more prone to acquiring a mixed PdGa/Pd_2_Ga phase, being in good agreement with the above XRD results.

X-ray photoelectron spectroscopy (XPS) tests were used to investigate the chemical composition and elemental valence states. As shown in Fig. 2c and d, the contents of Pd^0^ and Ga^0^ in PdGa/Co_SA_-NC reach 80.6% and 86.1%, respectively, being significantly higher than the corresponding values of 70.7% and 66.2% in PdGa/NC, respectively. Considering that metallic species are typically more active in electrocatalysis, this observation may imply a higher activity of PdGa/Co_SA_-NC than PdGa/NC.^41,42^ In addition, the high resolution XPS spectra of Co 2p (Fig. S7, SI) show that PdGa/Co_SA_-NC contains only Co^2+^ and no Co^0^ and Co^3+^, and simultaneously Co–N species has been detected in the N 1s spectra. It clearly demonstrates the formation of Co–N coordination by which elemental Co mainly exists in the form of single atoms in PdGa/Co_SA_-NC.^42,43^

### 2.2 Electrocatalytic tests in the EOR

**Fig. 3:**
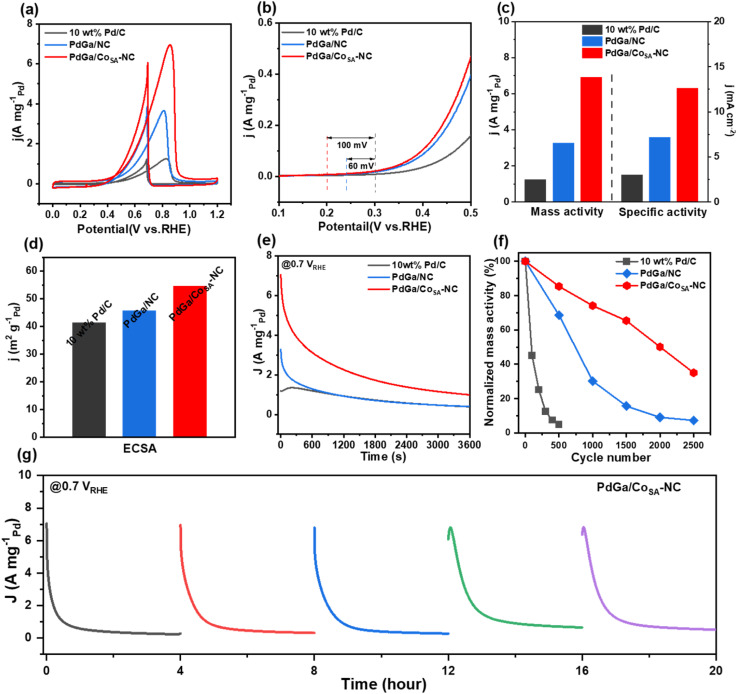
Catalytic EOR performance measurements. (a) Mass-normalized CV curves, (b) mass-normalized LSV curves, (c) MA and SA, (d) ECSA, (e) CA curves at 0.7 *V*_RHE_, (f) ADT tests of PdGa/Co_SA_-NC, PdGa/NC and commercial Pd/C in 1 M KOH + 1 M C_2_H_5_OH, and (g) long-term durability of PdGa/Co_SA_-NC. The catalyst reactivation and electrolyte replacement were done after every 4 hours.

The electrocatalytic EOR performances of commercial Pd/C, PdGa/NC and PdGa/Co_SA_-NC catalysts were evaluated using standard three-electrode tests conducted in N_2_-saturated 1 M KOH + 1 M CH_3_CH_2_OH electrolyte (Fig. 3a–d and S8, S9). In our work, the onset potential was identified as the potential where the current density measured in KOH + EtOH begins to exceed that in KOH alone, as determined from the CV curves in Fig. S8. Linear sweep voltammetry (LSV) polarization curves collected at a scan rate of 10 mV s^−1^ show that the onset EOR potential that occurred on PdGa/Co_SA_-NC is only 0.20 V, which is negatively shifted by 40 and 100 mV compared to those of PdGa/NC (0.24 V) and Pd/C (0.30 V), respectively, indicating more readily occurring EOR on PdGa/Co_SA_-NC. CO stripping voltammetry shows that the CO onset oxidation potential on PdGa/Co_SA_-NC is 20 and 70 mV lower than those on PdGa/NC and Pd/C, respectively, representing that PdGa/Co_SA_-NC has better anti-CO poisoning capability.^44,45^ The estimated electrochemical active surface area (ECSA) is 54.76 m^2^ g_Pd_^−1^ for PdGa/Co_SA_-NC, which is also higher than those of PdGa/NC (45.81 m^2^ g_Pd_^−1^) and Pd/C (41.35 m^2^ g_Pd_^−1^). In particular, the mass activity of PdGa/Co_SA_-NC reaches 6.93 A mg_Pd_^−1^, which is 2.1 and 5.6 times those of PdGa/NC (3.28 A mg_Pd_^−1^) and Pd/C (1.24 A mg_Pd_^−1^), respectively, and the specific activities follow the same trend. Additionally, the catalytic kinetics were further evaluated through Tafel slope and electrochemical impedance spectroscopy (EIS) (Fig. S10). PdGa/Co_SA_-NC exhibited the lowest Tafel slope (178 mV dec^−1^) and the smallest charge transfer resistance (30.0 Ω), indicating superior performance to both PdGa/NC and commercial Pd/C. These results suggest enhanced catalytic activity and improved reaction kinetics for the PdGa/Co_SA_-NC catalyst.^46,47^ We also compared the mass activity with the top-performing ones reported recently in Table S3, in which our catalyst is quite excellent.

The EOR durability of PdGa/Co_SA_-NC was studied by three methods. Firstly, in the chronoamperometry (CA) tests, the current–time (*i*–*t*) curves (Fig. 3e) were recorded at a constant voltage of 0.7 V for 3600 s in N_2_-saturated 1 M KOH + 1 M CH_3_CH_2_OH electrolyte. PdGa/Co_SA_-NC gives continuously higher current density with a slower current decrease rate than the other two catalysts throughout the test range. Secondly, we performed the CA tests 5 times by replacing the used electrolyte with a fresh one every 4 hours. Fig. 3g and S11 show that PdGa/Co_SA_-NC well retains the initial activity along this process, whereas PdGa/NC and Pd/C gradually deteriorate with increasing cycle number, and their 5th cycles provide only 39% and 18% of the initial current density, respectively. Thirdly, the accelerated durability testing (ADT) was conducted by applying multiple CV cycling in N_2_-saturated 1 M KOH+1 M CH_3_CH_2_OH solution. As shown in Fig. 3f and S12, Pd/C deactivates almost completely (only 4.8% activity remained) after 500 cycles. PdGa/NC is able to retain 30.2% of the initial activity after 1000 cycles, while after 1500 cycles, the retained activity is not more than 20.0%. In comparison, PdGa/Co_SA_-NC well retains 85.5% and 74.2% of the initial activity after 500 and 1000 cycles, respectively, and after 2500 consecutive cycles, there still is 35.1% activity left. We then investigated the microscopic morphology evolution of the PdGa/Co_SA_-NC catalysts. As shown in Fig. S13, following the ADT, PdGa nanocrystals in the PdGa/Co_SA_-NC catalyst exhibited only a slight size increase from 14.0 nm to 14.8 nm, and the single atom dispersion of Co sites remained unchanged. Therefore, the excellent structural stability, combined with high CO poisoning resistance, accounts for the outstanding durability of the PdGa/Co_SA_-NC catalyst. All the above observations clarify the superior activity and durability of PdGa/Co_SA_-NC in the electrocatalytic EOR, and the intrinsic mechanism was further investigated by *in situ* FTIR spectroscopy and DFT methods.

### 2.3 Reaction mechanism

**Fig. 4:**
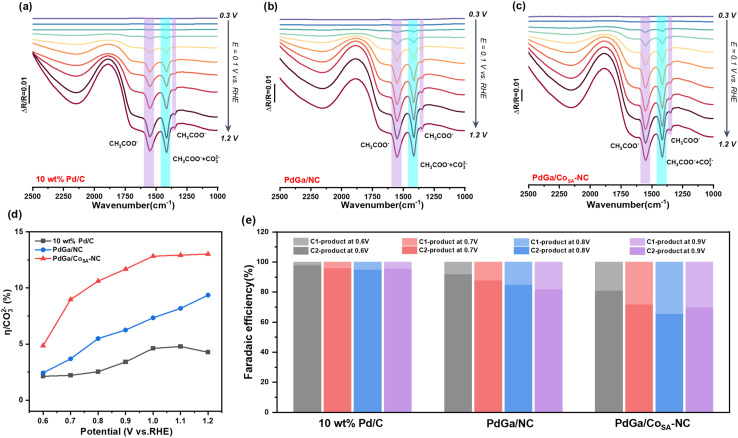
Electrochemical *in situ* FTIR spectra of (a) commercial Pd/C, (b) PdGa/NC and (c) PdGa/Co_SA_-NC in 1 M KOH + 1 M C_2_H_5_OH; (d) the selectivity (*η*) for complete ethanol oxidation to CO_3_^2−^ from 0.6 to 1.2 *V*_RHE_; (e) the faradaic efficiencies of the C1 pathway and C2 pathway from 0.6 to 0.9 *V*_RHE_ of catalysts.

Electrochemical *in situ* FTIR spectra were collected at the potentials ranging from 0.3 to 1.2 *V*_RHE_ with a spacing of 0.1 V. In Fig. 4a–c, the three characteristic peaks located at 1346, 1415 and 1550 cm^−1^ are attributed to the bending vibration of –CH_3_, and the symmetric and asymmetric stretching bonds of O–C–O in CH_3_COO^−^, respectively, which indicate the formation of C2 products. Interestingly, the three peaks are observed from 0.6 V on Pd/C, 0.5 V on PdGa/NC, and 0.4 V on PdGa/Co_SA_-NC, revealing the faster EOR kinetics on PdGa/Co_SA_-NC. Besides, the band of the possible C1 product CO_3_^2−^ is located at 1390 cm^−1^, which is overlapped with the peak at 1415 cm^−1^ assigned to acetate. We then roughly distinguish the presence of CO_3_^2−^ by a peak splitting. In Fig. S14, the CO_3_^2−^ relative spectral peak intensity of PdGa/Co_SA_-NC is significantly higher than those of Pd/C and PdGa/NC over the full range from 0.6 to 0.9 *V*_RHE_, suggesting that the PdGa/Co_SA_-NC catalyst may possess a higher C1-pathway selectivity in the EOR process. To more accurately determine the C1 route selectivity, subtractive spectroscopy was used to exclude the contribution of CH_3_COO^−^ at 1415 cm^−1^, by which the relative concentrations (*C*_R_) of CH_3_COO^−^ and CO_3_^2−^ and the C1-pathway selectivity *η* have been calculated (Fig. 4d and S15).^34,48–50^ In particular, *η* of PdGa/Co_SA_-NC is much higher than those of PdGa/NC and Pd/C over the potentials from 0.6 to 1.2 *V*_RHE_, implying that PdGa/Co_SA_-NC is very favorable to perform complete oxidation of ethanol. In addition, the oxidation products after long-term CA tests at different potentials were quantitatively analyzed by ^1^H NMR using the external standard curve method (Fig. S16 and S17), by which we further calculate the Faraday efficiencies (FEs). Fig. 4e shows that FEs of PdGa/Co_SA_-NC for C1 pathway are consistently better than those of PdGa/NC and Pd/C. The highest C1 selectivity on PdGa/Co_SA_-NC was acquired at the applied potential of 0.8 *V*_RHE_, which reaches 34.3%, being much higher than the corresponding values achieved on PdGa/NC (15.1%) and Pd/C (4.9%). The electrochemical test results and the *in situ* FTIR analyses suggest that, for the EOR, PdGa/Co_SA_-NC delivers a higher activity, durability and C1-pathway selectivity compared to PdGa/NC. It was deduced that the single atom Co_SA_-NC support may play an essential role for which we further conducted a DFT calculation study.

**Fig. 5:**
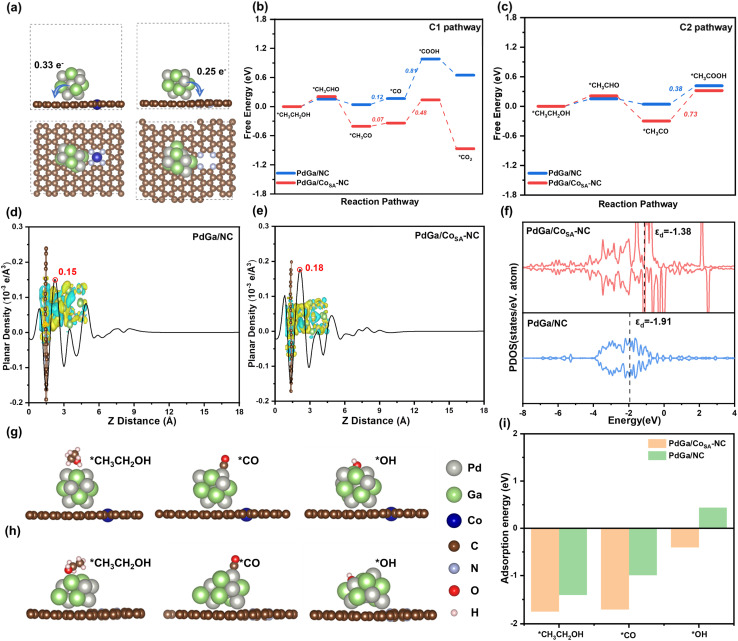
Theoretical calculation. (a) Optimized structures of PdGa/Co_SA_-NC and PdGa/NC (side view and top view); (b) free energy diagram of the C1 reaction pathway on PdGa/Co_SA_-NC and PdGa/NC; (c) free energy diagram of the C2 reaction pathway on PdGa/Co_SA_-NC and PdGa/NC. (d and e) Planar-averaged charge density difference (CDD) of PdGa/Co_SA_-NC and PdGa/NC. The insets show side views of CDD of PdGa/Co_SA_-NC and PdGa/NC. (f) The PDOS plots of PdGa/Co_SA_-NC and PdGa/NC; (g and h) adsorption configurations of key intermediates on PdGa/Co_SA_-NC and PdGa/NC (side view); (i) calculated adsorption energies of *CH_3_CH_2_OH, *CO and *OH on the surface of PdGa/Co_SA_-NC and PdGa/NC.

In our DFT calculations, two structures, the PdGa alloys with an atomic Pd : Ga ratio of 1 : 1 were constructed on Co_SA_-NC and NC substrates (Fig. 5a). Then the free energy diagrams along C1 and C2 EOR pathways were investigated (Fig. 5b, c and S18). For the C1–12e pathway, the C–C bond splitting of *CH_3_CO → *CO exhibits a lower energy barrier on PdGa/Co_SA_-NC (0.07 eV) than that on PdGa/NC (0.12 eV), suggesting that PdGa/Co_SA_-NC has a higher C1 pathway selectivity. Besides, the rate determining step (RDS) of the C1 path is a combination of *CO and *OH to produce *COOH, and its barrier of 0.48 eV on PdGa/Co_SA_-NC is distinctly lower than the value of 0.81 eV on PdGa/NC.^51–53^ This implies that PdGa/Co_SA_-NC is more favorable for the C1 pathway and is also better resistant to CO poisoning than PdGa/NC. In the C2 route, the RDS is *CH_3_CO oxidation to *CH_3_COOH, which exhibits a higher barrier on PdGa/Co_SA_-NC (0.73 eV) than on PdGa/NC (0.38 eV), implying that the C2 pathway occurs less frequently on PdGa/Co_SA_-NC.

The above observation clarifies that PdGa/Co_SA_-NC is more favorable for the C1 pathway and less favorable for the C2 pathway, and the opposite is true for PdGa/NC. We thus consider that the single atom Co dispersed Co_SA_-NC support in the former may play an important role, and allow for a deeper discussion. Bader charge analysis indicates that in PdGa/Co_SA_-NC, the PdGa alloy transfers 0.33 e^−^ to the Co_SA_-NC support, being more than the corresponding value of 0.25 e^−^ occurring in PdGa/NC. The planar-averaged charge density difference (CDD) plots (Fig. 5d and e) show that for both catalysts, a distinct peak appears at the interface of the PdGa alloy and the substrate, implying a substantial charge redistribution at this region. And compared to the charge density of 0.15 × 10^−3^ e A^−3^ for PdGa/NC, PdGa/Co_SA_-NC has a larger value of 0.18 × 10^−3^ e A^−3^. This observation matches well with the Bader charge calculation result that more electron transfers occur on PdGa/Co_SA_-NC, and both results reveal stronger interface interaction, thus more stable anchoring of PdGa nanoparticles on Co_SA_-NC than on the NC support.^54,55^ In addition, such electron interaction further leads to an upward shift of the d-band center of the PdGa alloy from −1.91 eV for PdGa/NC to −1.38 eV for PdGa/Co_SA_-NC (Fig. 5f). This shows that the transfer of electrons from the PdGa d-orbitals to the adsorbed species is faster because the d-band center is closer to the Fermi energy level.^21,23^ Besides, we also calculated the adsorption energies of *CH_3_CH_2_OH, *OH and *CO, three key species that could greatly affect the EOR pathway, and they all displayed significantly stronger adsorption on PdGa/Co_SA_-NC compared to PdGa/NC (Fig. 5g, h and S19). Therefore, for PdGa/Co_SA_-NC, due to the existence of the single atom Co in the NC support, the adsorption energies of the intermediate species are well optimized which accelerates the C–C bond breaking and *CO oxidation, and simultaneously inhibits the *CH_3_CO oxidation, thus leading to a higher C1 pathway selectivity. In addition, the charge transfer between PdGa alloy particles and Co single atoms greatly enhances the interfacial interaction force, and improves the anchoring strength of PdGa alloys on the support, thus significantly boosting the structural stability of PdGa/Co_SA_-NC. This, coupled with its high resistance to CO poisoning, results in superior electrochemical EOR durability.

## 3 Conclusion

In summary, we synthesized a PdGa/Co_SA_-NC catalyst and demonstrated that engineering a single atom Co dispersed support is an effective strategy for boosting the EOR performance of catalytic PdGa alloy centers. In the electrochemical EOR, PdGa/Co_SA_-NC achieves a mass activity of 6.93 A mg_Pd_^−1^ and a C1 path selectivity of 34.3% at 0.8 *V*_RHE_, which are 2.1 and 2.3 times those of the Co-free PdGa/NC counterpart, respectively. Additionally, it exhibited excellent long-term durability, retaining 74.2% and 35.0% of its initial activity after 1000 and 2500 cycles, respectively. DFT calculations reveal that PdGa alloy nanoparticles exhibit significantly enhanced electron transfer to the Co_SA_-NC support when compared to PdGa/NC, leading to a substantial increase in charge density at the interfacial region of PdGa/Co_SA_-NC. This charge redistribution further optimizes the adsorption energies of key intermediate species on Pd sites, promoting C–C bond cleavage and *CO oxidation for enhanced C1 selectivity as well as superior durability. This study offers valuable insights for the rational design of highly efficient catalysts for a range of crucial electrocatalytic processes.

## Author contributions

Chengming Huang conceived the project, performed data analysis, and wrote the original draft. Xia Chen, Wenjing Zhang, and Fangzheng Wang conducted data verification and curation. Yunchuan Tu performed data analysis. Jing Li reviewed and revised the manuscript and supervised the project. Zidong Wei provided resources.

## Conflicts of interest

The authors declare no competing financial interest.

## Supplementary Material

SC-016-D5SC05140A-s001

## Data Availability

The data that support the findings of this study are available in the paper and SI. Supplementary information is available. See DOI: https://doi.org/10.1039/d5sc05140a.
