# Supplementary material for: PdGa nanoalloys loaded on single atom Co dispersed nitrogen doped carbon for ethanol electrooxidation: improved C1 pathway selectivity and durability
Source: Chem Sci. 2025 Sep 23;16(42):20012–20. doi: 10.1039/d5sc05140a (PMC12483099; doi:10.1039/d5sc05140a)
Supplement: SC-016-D5SC05140A-s001 [file SC-016-D5SC05140A-s001.pdf]

Supplementary Information (SI) for Chemical Science.
This journal is © The Royal Society of Chemistry 2025

Supporting information for

PdGa nanoalloys loaded on single atomic Co dispersed nitrogen doped carbon for ethanol electrooxidation: improved C1 pathway selectivity and durability

Chengming Huang, Xia Chen, Wenjing Zhang, Fangzheng Wang, Yunchuan Tu, Jing Li,* and Zidong Wei**

State Key Laboratory of Advanced Chemical Power Sources, School of Chemistry and Chemical Engineering, Chongqing University, 401331 Chongqing, China

Experimental section

Chemicals

All chemicals were used as received without further purification. $Pd(acac)_2$, $Ga(acac)_3$, $Zn(NO_3)_2{\cdot}6H_2O$, $Co(NO_3)_2{\cdot}6H_2O$, KOH, and 2 methylimidazole were purchased from Aladdin Company. Methanol and ethanol were purchased from Sinopharm Chemical Reagent Ltd., China. Commercial Pd/C was purchased from Sigma. Deionized (DI) water was used in all experiments.

Synthesis of Zn/Co-ZIF

In the typical synthesis procedure, 1.98 g of $Zn(NO_3)_2{\cdot}6H_2O$ and 0.02 g of $Co(NO_3)_2{\cdot}6H_2O$ were dissolved in 100 mL of methanol, which was subsequently added into 100 mL of methanol containing 5 g of 2 methylimidazole with vigorous stirring for 24 h at room temperature. The mixed solution was then transferred to a Teflon lined stainless steel autoclave and kept at 120 °C for 12 hours. Finally, the resulting product was centrifuged, washed three times with methanol and dried under vacuum at 40 °C. The preparation method of Zn-ZIF was the similar to that of Zn/Co-ZIF, except that the Co precursor was not added.

Synthesis of PdGa/Co_{SA}-NC

Firstly, 1 g of Zn/Co-ZIF was dispersed in 50 mL of methanol by sonication, obtaining the solution A. Then, $Pd(acac)_2$ and $Ga(acac)_3$ were dispersed in 50 mL of methanol by sonication, obtaining the solution B. Solution A was mixed with solution B and stirred for 12 hours, then dried

by vacuum rotary evaporation at 50 °C. The resulting powder was placed in a tube furnace filled with 90% Ar/10% H_2 at a flow rate of 70 mL/min, heated up to 900 °C at a rate of 5 °C min^{-1}, maintained at this temperature for 2 h, and then naturally cooled to room temperature under a constant airflow throughout the process. The PdGa/Co_{SA}-NC catalyst was finally obtained. The preparation method of PdGa/NC was the similar to that of PdGa/Co_{SA}-NC, and the support was chosen as Zn-ZIF.

Characterizations

Transmission electron microscopy (TEM), scanning transmission electron microscopy (STEM) and energy-dispersive spectroscopy (EDS) measurements were conducted on a JEOL ARM200F electron microscope operated at 200 kV. All TEM samples were prepared by depositing a drop of diluted ethanol suspension on a copper grid coated with carbon film. X-ray diffraction (XRD) data were collected on a XRD-6000 using Cu Kr radiation (λ=1.5418 Å) at a step rate of 2° min^{-1}. Before measurements, the samples were degassed in vacuo at 180 °C for 8 h. X-ray photoelectron spectra (XPS) were recorded on a Thermo Scientific K-Alpha, and the spectra were calibrated with the C1s peak at 284.8 eV as an internal standard. The elemental contents were analyzed by ICP-OES (Inductively coupled plasma optical emission spectrometer) on a Thermo Scientific iCAP 6300 Duo.

Calculation of the lattice parameters of specimens

The crystallite size of specimens was calculated by the Debye-Scherrer formula：

$$D = \frac{k\lambda}{\beta\cos\theta}$$

where D is crystallite size (nm), k is Scherrer constant (0.89), θ is the Bragg angle, λ and β present the X-ray wavelength (λ = 1.5418 Å), and line broadening at half the maximum intensity (FWHM), respectively.

The interplanar spacing of specimens was calculated by Bragg's law：

$$d = \frac{\lambda}{2\sin\theta}$$

where: d is lattice space (Å); λ and θ denote the X-ray wavelength (λ = 1.5418 Å) and Bragg angle, respectively.

The lattice strain of the specimens was calculated as follows:

$$L = 1/2\mathrm{d}$$

$$L_2\ \mathrm{strain\%} = (L_1 - L_2)/L_1$$

where L denotes the lattice parameter and d is the interplanar spacing of the selected crystal facet. L_2% represents the percentage change of the lattice parameter of the PdGa/Co_{SA}-NC when compared to PdGa/NC. L_1 and L_2 are the lattice parameters of PdGa/NC and PdGa/Co_{SA}-NC, respectively.

Electrochemical measurements

The electrochemical performances of the catalysts were characterized by cyclic voltammetry (CV) and linear sweep voltammetry (LSV) techniques. All electrochemical experiments were detected by electrochemical workstation (Versa STAT 3F). To fabricate the working electrodes, the as-synthesized catalyst (2 mg) was dispersed in 600 μL of ethanol along with 5 μL of Nafion solution (5 wt%), followed by ultrasonication for 60 minutes to form a homogeneous ink. Then, 10 μL of the resulting suspension was drop-cast onto a glassy carbon rotating disk electrode (RDE, 5 mm in diameter) using a micropipette. A standard three-electrode system was employed for all electrochemical measurements, comprising an Hg/HgO reference electrode, a carbon rod counter electrode, and a catalyst-loaded rotating disk electrode (RDE) as the working electrode. Before electrochemical measurements, all electrodes were pretreated by cyclic voltammetry (CV) between 0 V to 1.2 V at a sweep rate of 50 mV s^{-1} for 60 cycles to remove surface contamination. To test the ethanol oxidation performances, the CV tests were performed in N_2-purged mixed solution of 1 M KOH and 1 M CH_3CH_2OH at a scan rate of 50 mV s^{-1}.

The CO stripping were conducted in N_2-saturated 1 M KOH solution which was purged with CO before the measurement at 50 mV/s. The electrochemical surface area (ECSA) of Pd was calculated by the following equation:

$$ECSA = \frac{Q}{0.405 \times [W_{Pd}]}$$

where W_{Pd} is Pd loading (mg · cm^2) on the electrode, Q is the Coulombic charge by integrating the peak area of the reduction of PdO (mC), and 0.405 represents the charge required for the reduction of a PdO monolayer (mC · cm_{Pd}^{-2}).

For accelerated durability test, two methods, chronoamperometry (CA) and cyclic voltammetry (CV) were investigated. In CA test, the time-dependent current density was recorded under a static voltage of 0.7 V (versus RHE). In CV test, the electrode was scanned between 0 and 1.2 V (versus RHE) for 2500 continuous cycles in the mixed solution of 1M KOH and 1 M CH_3CH_2OH.

In situ Fourier transform infrared spectroscopy (FTIR)

The electrochemical in-situ Fourier Transform-Infrared (FTIR) reflection spectroscopy measurements were recorded using the Nexus 870 spectrometer (Nicolet) equipped with a liquid nitrogen-cooled MCT-A detector. A calcium fluoride window and an in situ EC-IR thin-layer cell were employed in the test. Spectra were acquired at continuous stepped potentials from 0.1 V to 1.2 V vs. RHE, a resolution of 8 cm^{-1}. The resulting spectra were recorded as the relative change in reflectivity and calculated as follows:

$$\frac{\Delta R}{R}=\frac{R\ (E_S)\ -R(E_R)}{R(E_R)}$$

where $R(E_S)$ and $R(E_R)$ are the single-beam spectra collected at the sample potential E_S and reference potential E_R, respectively.

η was calculated to evaluate the selectivity of ethanol oxidation to CO_3^{2-}, as follows:

$$\eta=\frac{\left(CO_3^{2-}\right)/2}{\left(CO_3^{2-}\right)/2+CH_3COO^-}$$

Calculation of C1-pathway selectivity

The yield of possible EOR product can be conducted by off-line nuclear magnetic resonance (NMR, Bruker, 600 MHz) and Faraday formula. Firstly, a long time i-t test has been conducted to collect the product to be measure. The concentration of as-produced CH_3COO^- can be calculated by the standard curve. Each electrolyte was N_2-saturated 1.0 M KOH solution containing 1.0 M CH_3CH_2OH. 50 mL electrolyte was electrolyzed by chronoamperometry with different catalysts to accumulate the total charge (Q). Finally, based on the Faraday formula, the Faradic efficiency of as-produced CH_3COO^- can be calculated as follow:

$$FE_{C2}=\frac{e\times n\times F}{Q}$$

Where e is the number of electrons transferred to CH_3COOH (e = 4), n is the total moles of CH_3COOH, F is the Faraday constant (96485.33 C mol^{-1}), and Q is the total charge. Finally, the possible FE of C1 product can be obtained by 1-FE_{C2}.

DFT calculations

All calculations in this work were performed by the density functional theory (DFT) within the Vienna ab initio simulation package (VASP). The ion–electron interaction was described with the projector augmented wave (PAW) method. Electron exchange-correlation was represented by the functional of Perdew, Burke, and Ernzerhof (PBE) of generalized gradient approximation (GGA). A cutoff energy of 450 eV was used for the plane-wave basis set (2×2×1) Monkhorst–Pack k-point meshes were used for calculations. In all of the calculations, the convergence criterion of the electronic structures was set to 10^{-5} eV, and the atomic positions were allowed to relax until the forces were less than 0.03 eV/Å. To separate the slab from its periodic images and to avoid spurious interactions, a vacuum height of 16 Å along the vertical direction was selected. The formation energy for metal nanoparticles is defined as follow:

$E_f = E_{tot} - E_{slab} - E_{metal}$

Where E_{tot} is the total energy of support and metal nanoparticle, E_{slab} is the energy of the clean support alone, and E_{metal} is the energy of metal nanoparticle.

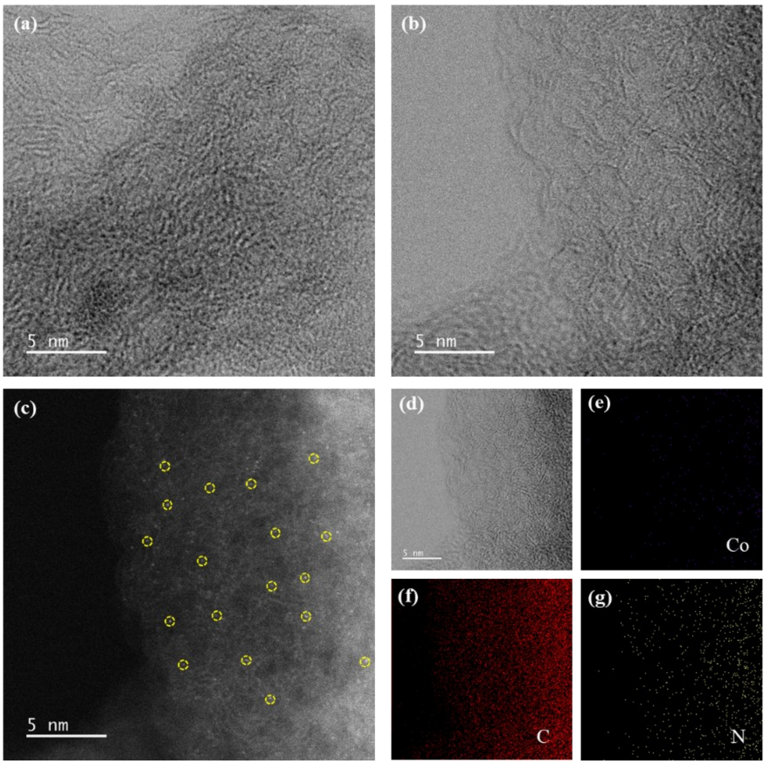

Figure S1. TEM, HAADF-TEM image and corresponding elemental maps of Co_{SA}-NC support.

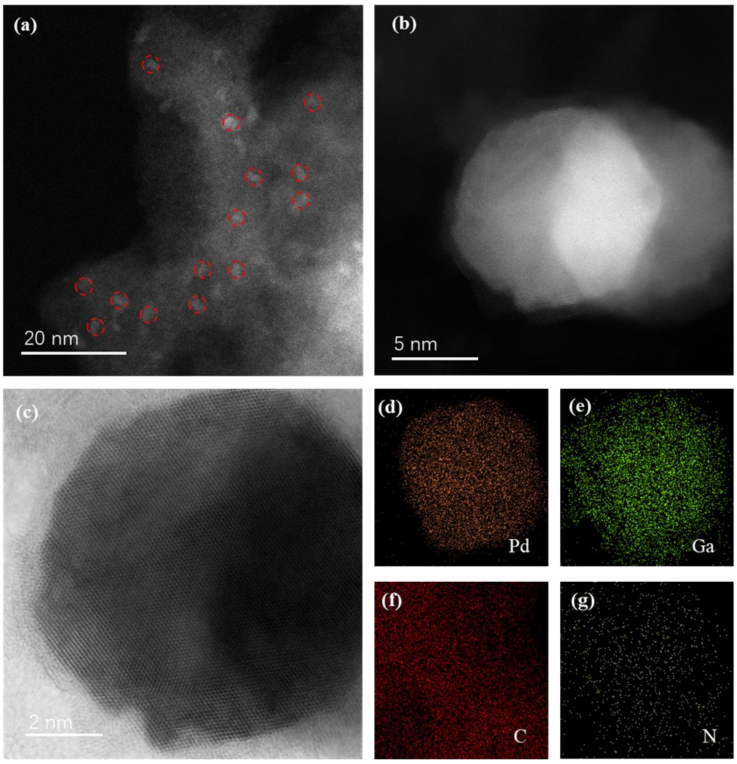

Figure S2. TEM, HAADF-TEM image and corresponding elemental maps of PdGa/Co_{SA}-NC.

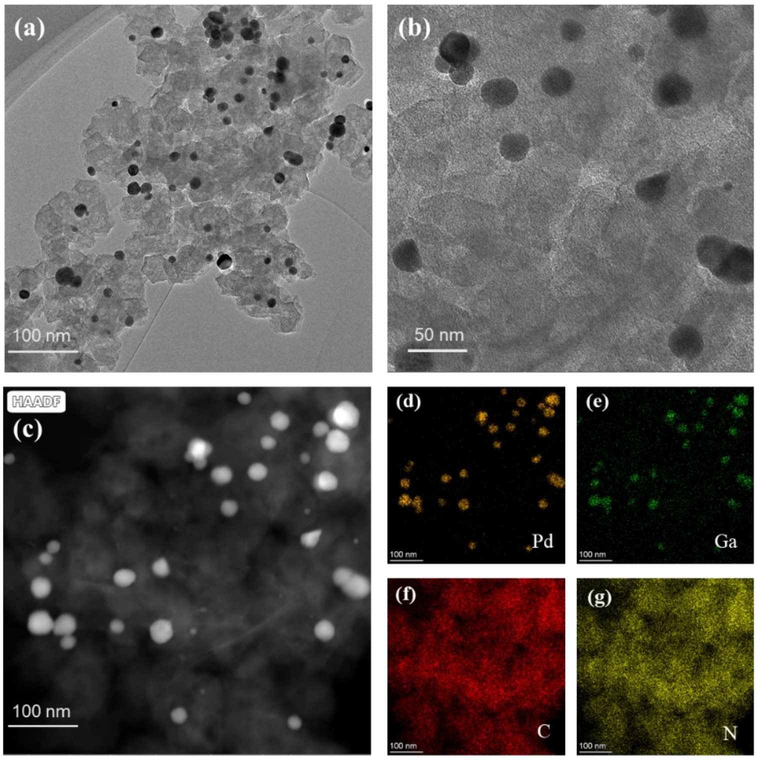

Figure S3. TEM, HAADF-TEM image and corresponding elemental maps of PdGa/NC.

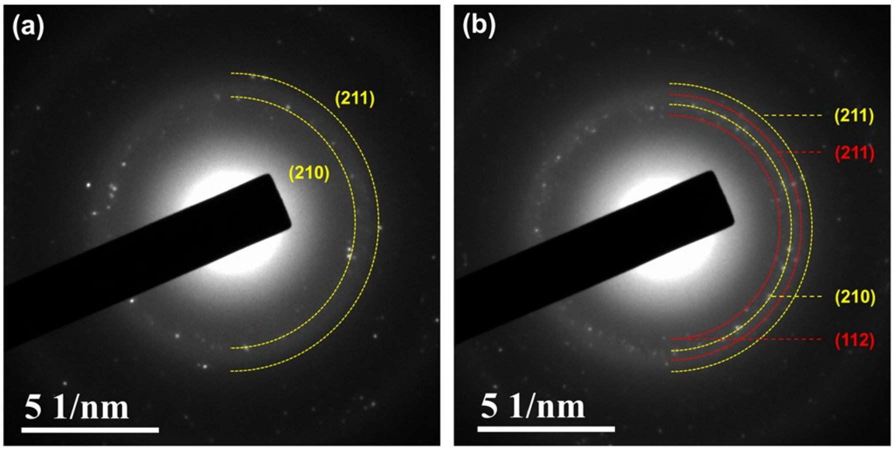

Figure S4. SAED patterns of (a) PdGa/Co_{SA}-NC and (b) PdGa/NC. Yellow and red represent the diffraction planes of PdGa and Pd_2Ga, respectively.

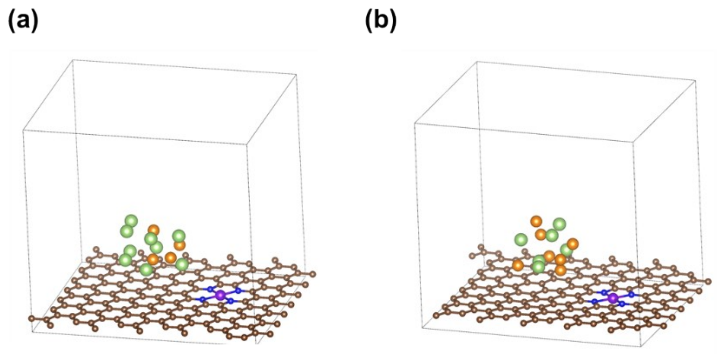

Figure S5. Optimized structures of (a) $PdGa_2$, (b) PdGa and (c) Pd_2Ga on Co_{SA}-N-C support obtained by DFT calculations, respectively.

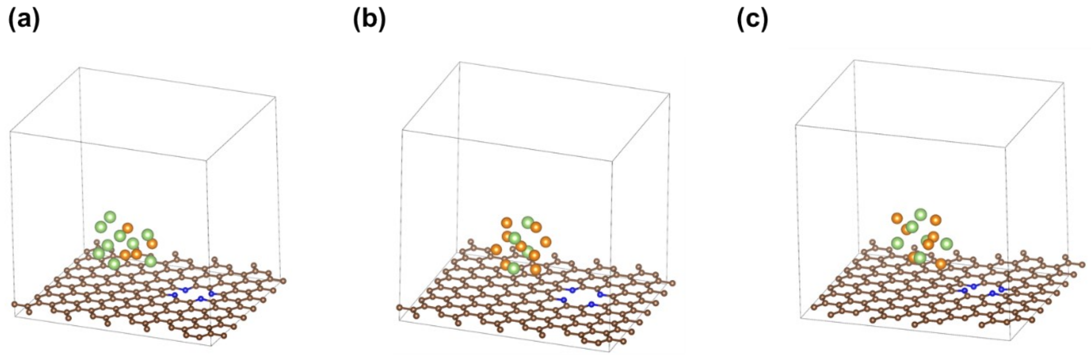

Figure S6. Structures model plots of (a) $PdGa_2$, (b) PdGa and (c) Pd_2Ga on N-C support obtained by DFT calculations, respectively.

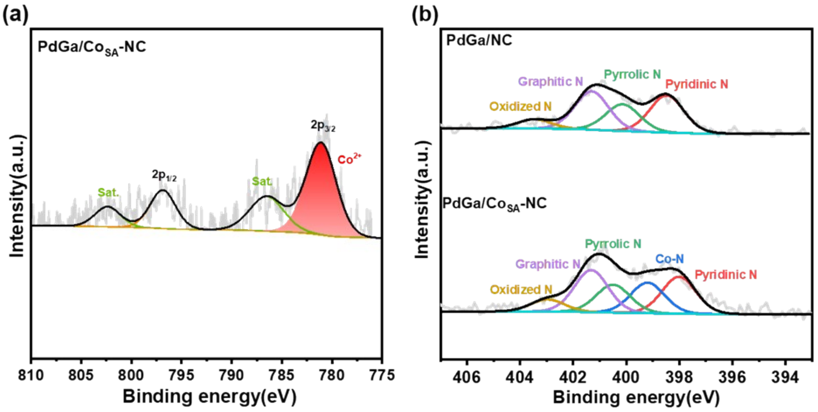

Figure S7. (a) Co 2p XPS spectra of PdGa/Co$_{SA}$-NC; (b) N 1s XPS spectra of PdGa/NC and PdGa/Co$_{SA}$-NC.

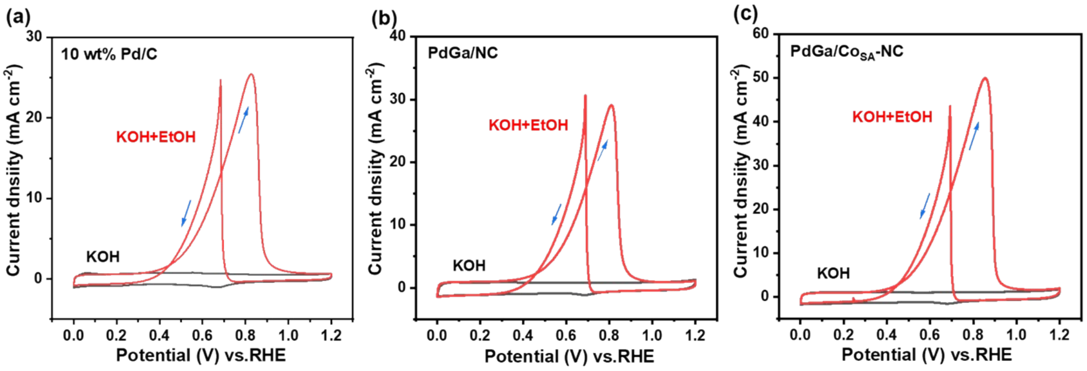

Figure S8. CVs of the catalysts in N_2-saturated 1 M KOH with and without 1 M C_2H_5OH. (a) commercial Pd/C, (b) PdGa/NC, (c) PdGa/Co_{SA}-NC.

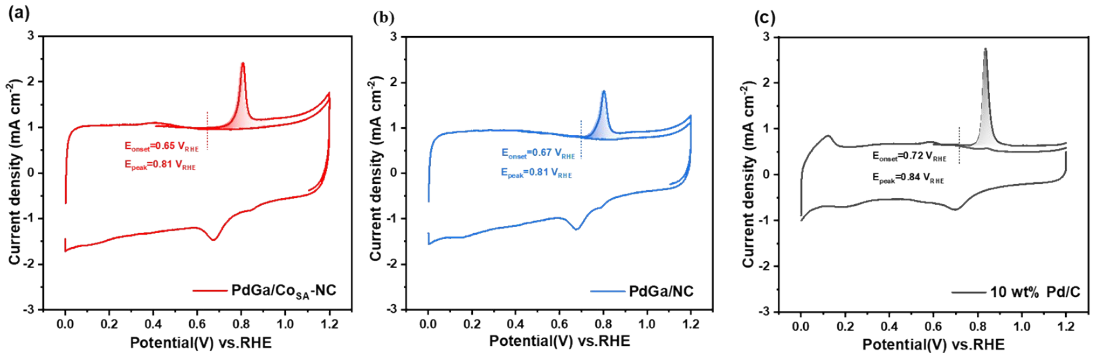

Figure S9. CO stripping voltammograms of (a) PdGa/Co_{SA}-NC, (b) PdGa/NC, (c) commercial Pd/C in 1 M KOH at a sweep rate of 50 mV/s.

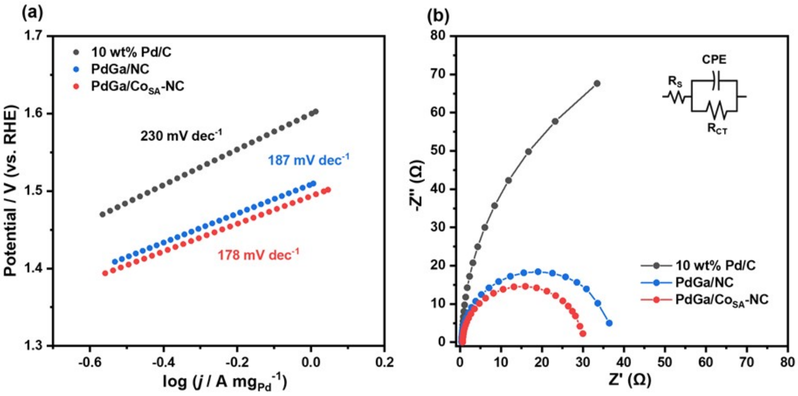

Figure S10. (a) Mass activity Tafel plots; (b) Nyquist plots recorded at 0.7 V_{RHE} for different catalysts in 1 M KOH + 1 M C_2H_5OH solution.

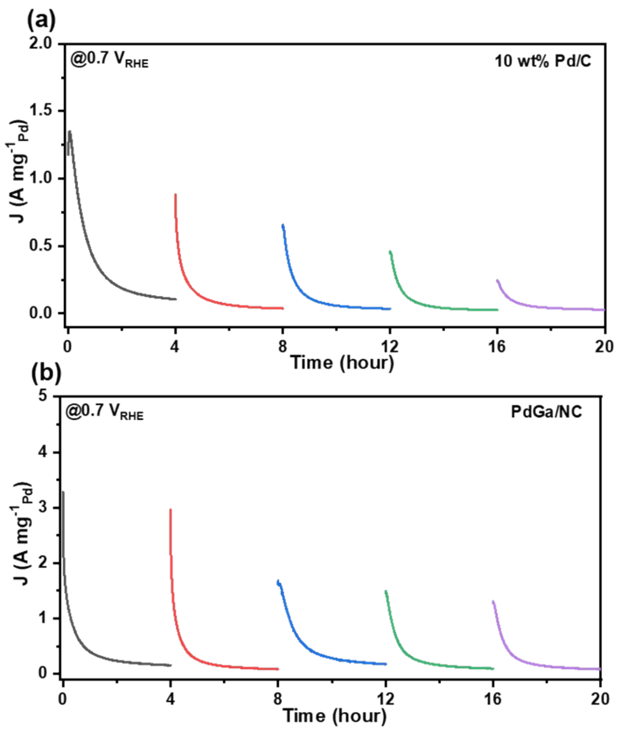

Figure S11. Long-term durability of (a) commercial Pd/C and (b) PdGa/NC. The catalyst reactivation and electrolyte replacement after every 4 hour.

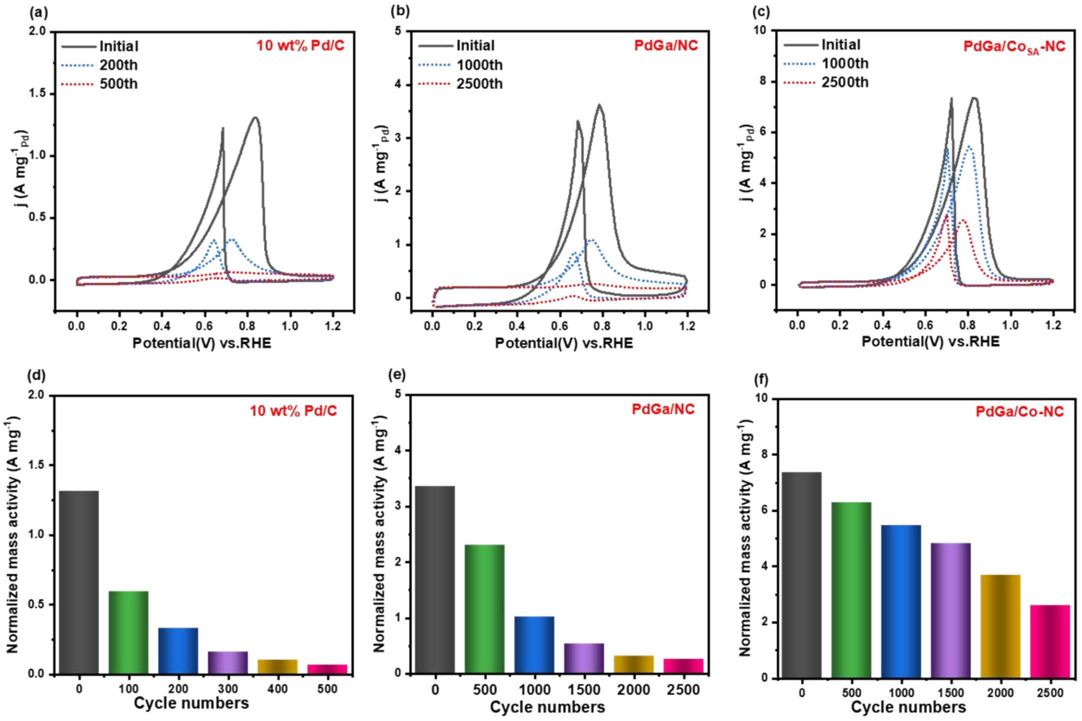

Figure S12. CVs and Mass activity of (a) commercial Pd/C, (b) PdGa/NC and (c) PdGa/Co_{SA}-NC in 1 M KOH + 1 M CH_3CH_2OH solution before and after the 500 or 2500 cycles.

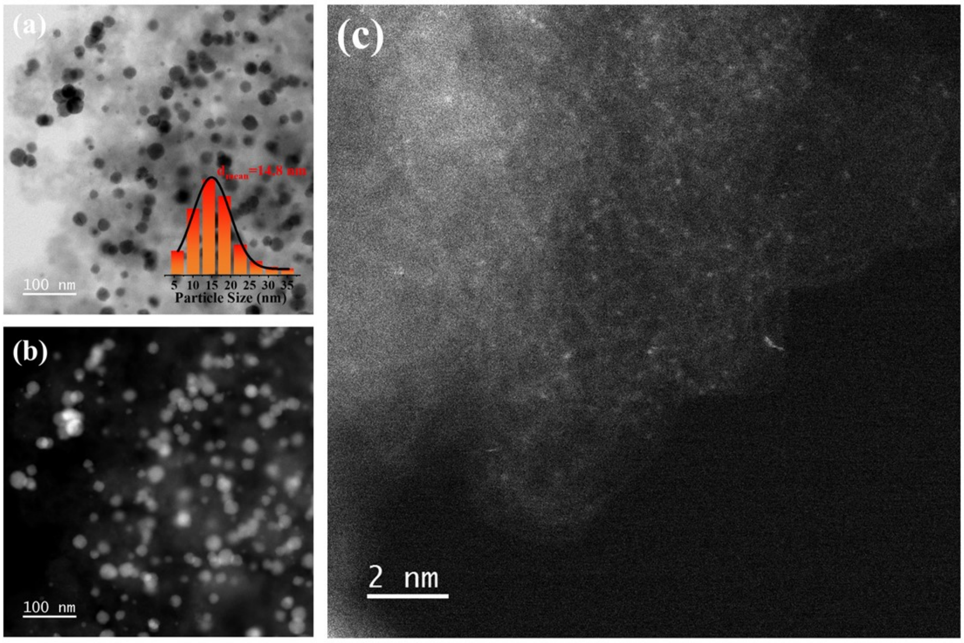

Figure S13. TEM, HAADF-STEM image and size distribution histogram of PdGa/Co_{SA}-NC catalyst after ADT.

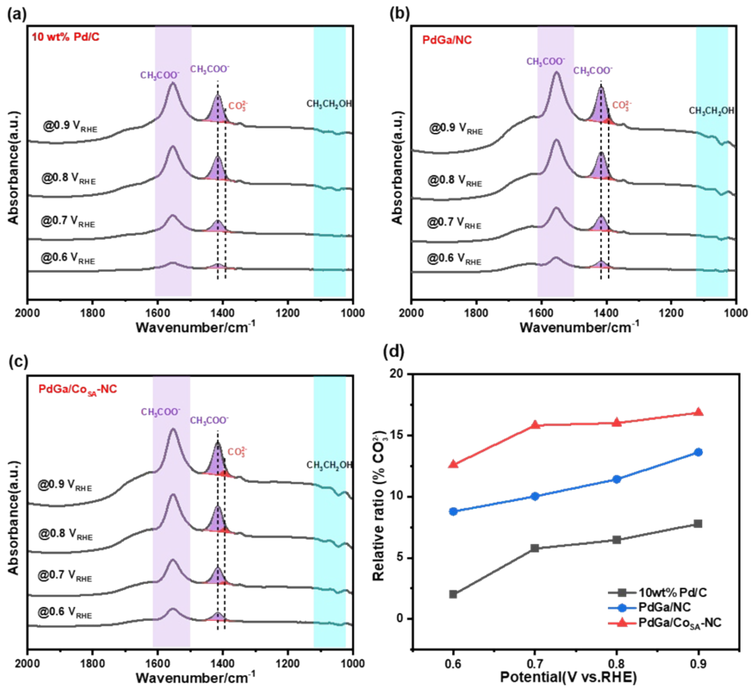

Figure S14. In situ FTIR spectra varied from 0.6 to 0.9 V versus RHE in 1 M KOH + 1 M ethanol of (a) commercial Pd/C, (b) PdGa/NC and (c) PdGa/Co$_{SA}$-NC; (d) The relative peak area ratios of CO_3^{2-} from 0.6 to 0.9 V_{RHE}.

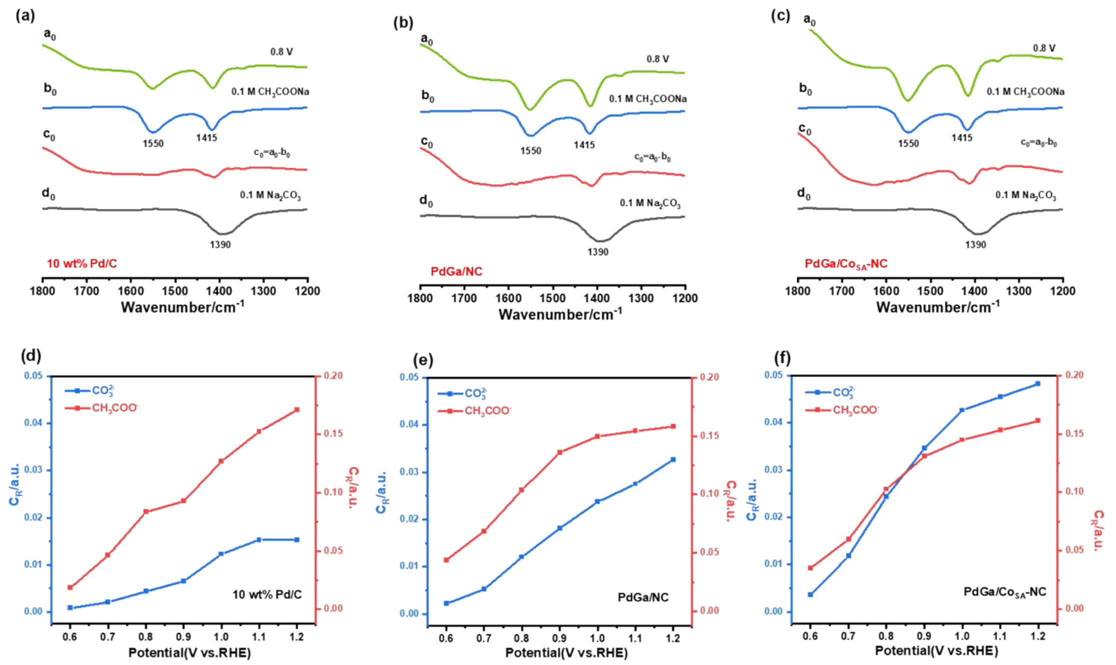

Figure S15. Identification of carbonate as products of ethanol oxidation on (a) commercial Pd/C, (b) PdGa/NC and (c) PdGa/Co_{SA}-NC at 0.8 V_{RHE}; (d-f) Potential dependence of the relative concentration (C_R) of CH_3COO^- and CO_3^{2-} for catalysis.

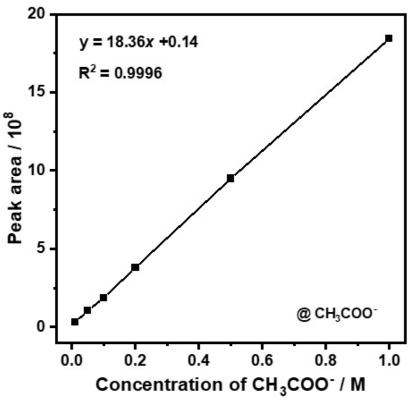

Figure S16. The external standard curves of CH_3COO^- is used to determine the concentrations in the corresponding residual electrolyte by ^{1}H NMR.

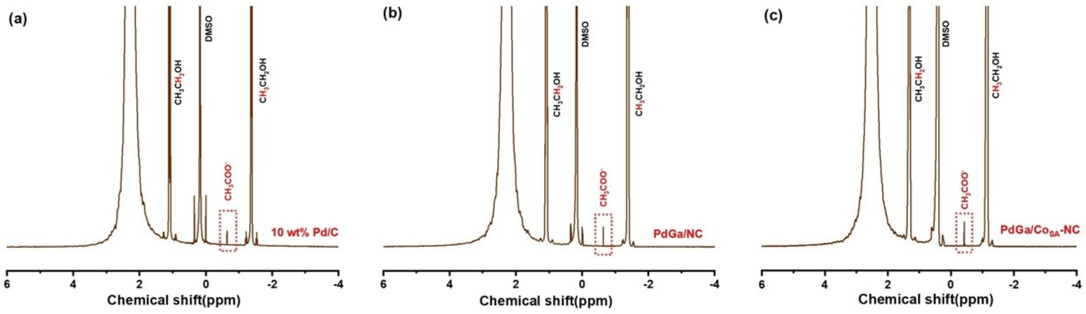

Figure S17. Typical ^{1}H nuclear magnetic resonance (NMR) spectra of (a) commercial Pd/C, (b) PdGa/NC and (c) PdGa/Co_{SA}-NC in 1 M KOH + 1 M ethanol solution at 0.8 V_{RHE}.

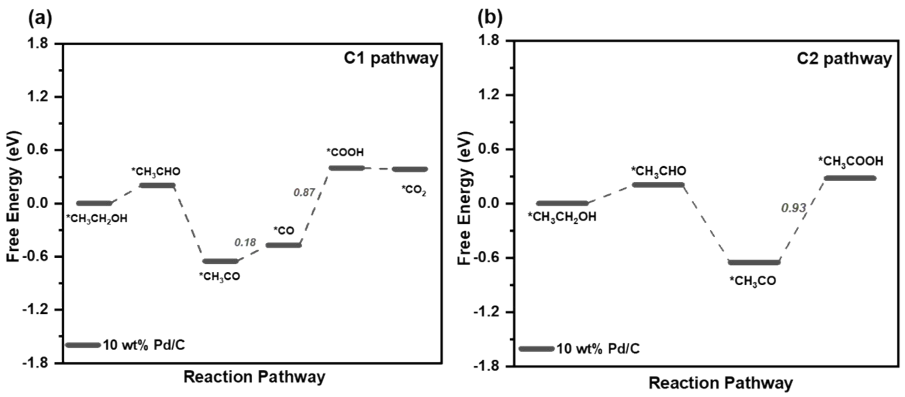

Figure S18. Free energy diagram of C1 and C2 reaction pathway on commercial Pd/C.

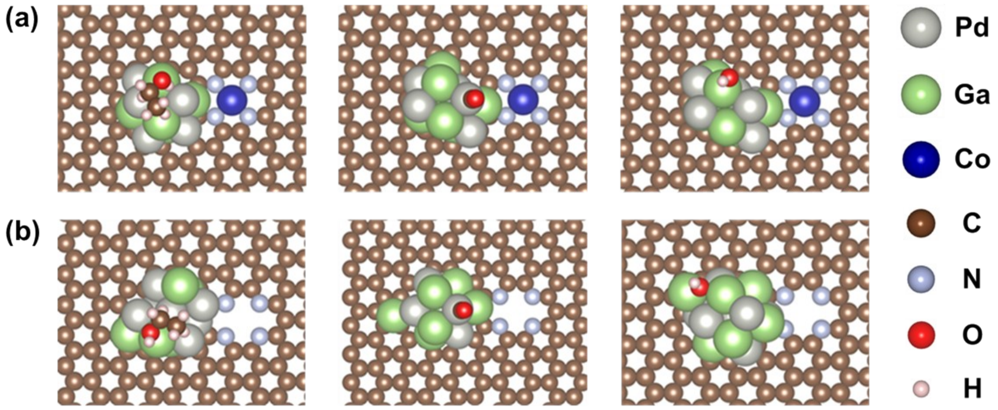

Figure S19. Adsorption configurations of key intermediates on (a) PdGa/Co_{SA}-NC and (b) PdGa/NC (Top view).

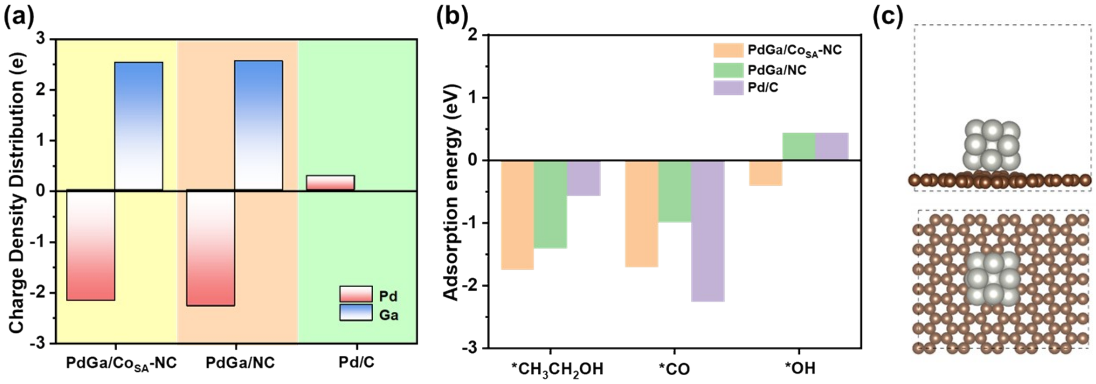

Figure S20. DFT calculation results. (a) The charge density distributions; (b) Calculated adsorption energies of *CH_3CH_2OH, *CO and *OH on the surface of PdGa/Co_{SA}-NC, PdGa/NC and Pd/C; (c) Optimized structures Pd/C (Slide view and top view).

As revealed by the calculated charge density distribution in Figure S19a, pure Pd exhibits a positive charge. Upon alloy formation, however, the Pd atoms in both PdGa alloys become more negatively charged, indicating electron transfer from Ga to Pd. This alters the electron density on the Pd surface. The adsorption energies calculated in Figure S19b demonstrate that compared to pure Pd, both alloys exhibit stronger adsorption of *CH_3CH_2OH but weaker adsorption of *CO. This indicates that PdGa alloy formation facilitates the EOR by enhancing reactant adsorption while simultaneously mitigating CO poisoning. Notably, the two alloys show divergent adsorption trends for *OH, a key reactant for *CO removal. PdGa/NC displays similarly weak *OH adsorption capacity as pure Pd, whereas PdGa/Co_{SA}-NC exhibits significantly stronger *OH adsorption. Thus single atomic Co is essential for this enhancement. Since its role is established in the main manuscript, our discussion here focuses exclusively on the role of Ga.

In summary, Ga plays a critical role in the alloys by donating electrons to Pd, thereby modifying its electronic structure. This electronic modulation promotes *CH_3CH_2OH adsorption while suppressing *CO binding.

Table S1. ICP-OES results of catalysts.

Catalysts	Pd loading (wt. %)	Ga loading (wt. %)	Co loading (wt. %)	Zn loading (wt. %)
Pd/C	10	--	--	--
PdGa/NC	3.9	3.5	--	--
PdGa/Co_{SA}-NC	3.9	3.4	1.4	--

Table S2. A summary of lattice parameters of catalysts.

Catalysts	2θ / degree PdGa (210)	Crystalline size (nm)	Interplanar spacing (nm)	Strain (%)
PdGa/NC	41.37	11.8	0.2182	--
PdGa/Co_{SA}-NC	41.68	10.4	0.2167	0.69

Table S3. Summary of the EOR performance of Pd-based catalysts reported in recent years.

Samples	Electrolyte	mA (A/mg)	C1 selectivity	Stability after ADT	Reference
PdGa/Co_{SA}-NC	1M KOH + 1M Ethanol	6.92	34.3%	74.2% (1000)	**This work**
				35.0% (2500)	**This work**
Pt/α-PtOx/WO_3	0.1M NaOH + 0.5M Ethanol	2.76	21.9%	/	Adv. Funct. Mater. **2021**, 31, 2100982
m-PtPb NSs	1M KOH + 0.1M Ethanol	1.23	5%	/	Angew. Chem. Int. Ed., **2023**, 24, e202305158
Pd-Au HNS/C	0.1M KOH + 1M Ethanol	8.0	33.2%	/	Adv. Energy Mater., **2021**, 11, 2100187.
PdAgSn/PtBi HEA NPs	1M KOH + 1M Ethanol	3.39	/	/	Angew. Chem. Int. Ed. **2023**, 135, e202304510
PdBi NS	1M KOH + 1M Ethanol	6.60	19.7%	30.8% (1000)	Appl. Catal. B: Environ. **2023**, 328, 122521
H-Ga1Pdene	1M KOH + 1M Ethanol	10.34	56.6%	67.3% (1000)	Appl. Catal. B. **2024**, 342, 123377
$L1_2$ PdCuSn	1M KOH + 1M Ethanol	6.22	/	/	Adv. Mater. **2022**, 34 , 2106115
Pd/Co@N-C	1M KOH + 1M Ethanol	7.06	/	84.2% (500)	Nat. Commun. **2023**, 14, 1346
PdAg SSs	1M KOH + 1M Ethanol	5.27	22.7%	/	Chem. Eng. J. **2024**, 495, 152536
$Pd_{1.84}Sn@SnO_x$	1M KOH + 1M Ethanol	0.5	/	98% (1500)	Adv. Mater. **2024**, 2415362
RhCuBi TMEs	1M KOH + 1M Ethanol	1.11	43.3%	/	Adv. Energy Mater., **2024**, 14, 2400112
Rh_7Pt_1 PBML	1M KOH + 1M Ethanol	1.77	100%	/	Energy Environ. Sci., **2024**, 17, 2219-2227
r-Pt/$Pd_{20}Sb_7$ NPs/C	0.5M NaOH + 0.5M Ethanol	3.97	72.4%	83.7% (2000)	Nature Nanotechnology, **2024**, 19, 1306-1315.
